# Checklist for the qualitative evaluation of clinical studies with particular focus on external validity and model validity

**DOI:** 10.1186/1471-2288-6-56

**Published:** 2006-12-11

**Authors:** Gudrun Bornhöft, Stefanie Maxion-Bergemann, Ursula Wolf, Gunver S Kienle, Andreas Michalsen, Horst C Vollmar, Simon Gilbertson, Peter F Matthiessen

**Affiliations:** 1 Chair in Medical Theory, Witten/Herdecke University, Gerhard-Kienle-Weg 4, D – 58313 Herdecke, Germany; 2 PanMedion Foundation, Bergstrasse 89, CH – 8032 Zürich, Switzerland; 3 Institute for Complementary Medicine, University of Berne, Imhoof-Pavillon, Inselspital, CH – 3010 Bern, Switzerland; 4 Institute for Applied Epistemology and Medical Methodology (IFAEMM), Schauinslandstr. 6, D – 79189 Bad Krozingen, Germany; 5 Dept. for Internal and Integrative Medicine, Kliniken Essen-Mitte, Am Deimelsberg 34a, D – 45276 Essen, Germany; 6 Competence Centre for General Practice and Outpatients' Health Care, Witten/Herdecke University, Alfred-Herrhausen-Str. 50, D – 58448 Witten, Germany; 7 Ruhrhöhenweg 2, D-45527 Hattingen, Germany

## Abstract

**Background:**

It is often stated that external validity is not sufficiently considered in the assessment of clinical studies. Although tools for its evaluation have been established, there is a lack of awareness of their significance and application. In this article, a comprehensive checklist is presented addressing these relevant criteria.

**Methods:**

The checklist was developed by listing the most commonly used assessment criteria for clinical studies. Additionally, specific lists for individual applications were included. The categories of biases of internal validity (selection, performance, attrition and detection bias) correspond to structural, treatment-related and observational differences between the test and control groups. Analogously, we have extended these categories to address external validity and model validity, regarding similarity between the study population/conditions and the general population/conditions related to structure, treatment and observation.

**Results:**

A checklist is presented, in which the evaluation criteria concerning external validity and model validity are systemised and transformed into a questionnaire format.

**Conclusion:**

The checklist presented in this article can be applied to both planning and evaluating of clinical studies. We encourage the prospective user to modify the checklists according to the respective application and research question. The higher expenditure needed for the evaluation of clinical studies in systematic reviews is justified, particularly in the light of the influential nature of their conclusions on therapeutic decisions and the creation of clinical guidelines.

## Background

It is known that clinical studies can generate discordant results. This observation is addressed scientifically in various ways. Deviant study results may be understood as an expression of spreading or scattering from a supposed true value (whereas deviation depends on the precision of the methods). An alternative approach is to explain differences not statistically but by way of content [1]. In considering individual studies, there should be an estimate to what extent the study conclusions are distorted by systematic factors of bias. Here the focus lies usually on so called internal validity, the comparability of test and control groups. (Detailed definitions of internal validity and other validity categories are given in the methods section). When assessing internal validity a differentiation is made between the following factors:

◆ Selection bias: differences between test and control population regarding their *structural *composition, e.g. in terms of age, gender, duration and severity of illness and others.

◆ Performance bias: differences in the *treatment *apart from the intervention tested, e.g. more contact, attention or efforts in the verum group.

◆ Detection bias: differences in *observation *of outcome parameters, e.g. due to inadequate blinding and respective expectations by assessors, due to training effects or others.

◆ Attrition bias: related to differences in dropouts between test and control group.

The goal is to gain the largest possible level of structural, treatment-related and observational similarity between test and control groups through randomisation and blinding, with a subsequent evaluation following the "intention to treat" (ITT) principle [1-3]. Studies with relative good avoidance of selection, performance, attrition and detection bias, in relation to the test and control populations, are classified as internally valid. Scoring systems have been developed to support the evaluation of internal validity (e.g., the Jadad Score) [4-6] and assessment criteria of internal validity are also reflected in the EBM hierarchy of study types [7-9]. In contrast, aspects of external validity that refer to the comparability between the study population and the general population of interest are often neglected in quality assessment and are usually not considered as having a possible distortive effect on an article's conclusion [10,11]. Rothwell stated in 2005 [11]: "There is concern among clinicians that external validity is often poor [...]. Yet researchers, funding agencies, ethics committees, the pharmaceutical industry, medical journals, and governmental regulators alike all neglect external validity, leaving clinicians to make judgments. However, reporting of the determinants of external validity in trial publications and systematic reviews is usually inadequate [...]."

Factors that can lower the representativeness of a study population and thus the external validity are for example:

◆ Process of consenting: patients who give their consent to participate have been shown to differ largely in severity of illness and other parameters to those who do not give their consent [12,13].

◆ Consenting and selection criteria: Emmerich et al. [14] interpret the fact that only 7–8% of possible study participants were included in a study in that way that the study population was highly selected, well motivated with good levels of compliance and better probable outcomes than the "real-life" patients. The most frequent exclusion criteria were relative contraindications to the study intervention and refusal of participants.

◆ Patients' preferences: Protheroe et al. [15] showed that the discrepancy between clinical guidelines and their practical application becomes larger when patient preferences are considered. According to their decision analysis only 60% of patients with atrial fibrillation had preferred anticoagulation, which was far less than those who would have been recommended by guidelines (up to 90%). When interpreting data on patients' preferences one should consider that answers in questionnaires or interviews are often discordant with actual decisions.

◆ Furthermore, commonly neglected factors that limit the validity of study results, according to Rothwell [11], are as varied as differences in health care systems, national characteristics and regulations, characteristics of the participating centres and the level of physicians' specialisation (for example, being limited to "special care units").

Regarding such contextual differences one should also distinguish, on the one hand, services' ability to deliver and, on the other hand, clients' uptake and potential to benefit.

◆ Other factors include the choice of outcomes: surrogate parameters, e.g. laboratory values instead of clinical values, and relevant parameters for the patient (general and mental health, emotional balance, vitality and quality of life), all of which are seldom charted in randomised controlled trials (RCTs).

Rothwell suggests that there should be a stronger consideration of external validity criteria in the evaluation of clinical studies, even in guidelines such as the CONSORT [16] or Cochrane Collaboration guidelines [2]. This issue was taken up by Glasgow and colleagues [17] in 2005. Concrete proposals for assessing generalisability in trials of health care interventions were made by Bonell et al. in 2006 [18].

The tools required to evaluate external validity are, in principle, not new – the relevant criteria have been used in methodology lectures for medical students, and are found in many guidelines for the evaluation of clinical studies. It seems, however, that there is a deficit in both the awareness of the actual necessity for this evaluation process and in the actual application of the assessment criteria.

With this article, we present a checklist that encompasses the most important quality assessment criteria regarding external validity and model validity criteria. These criteria have been systematised and have been formulated in operable questions.

## Methods

The checklist has been developed by listing the most commonly used assessment criteria for clinical studies [2-4,9,11,16,19-38] and by using specific criteria lists for individual applications. These include, for example: surgical interventions [39], so-called practical clinical studies which are characterised particularly by a larger amount of heterogeneity of population, intervention and outcome criteria [40], observational studies [41], single case analyses of oncology patients [28], the aforementioned criteria regarding external validity published by Rothwell [11], and model validity published by Wein [37], and our own assessment criteria: We extrapolated key elements from internal validity to external validity, adapting them where necessary. We integrated questions from the above mentioned lists into the scheme of external validity and added criteria derived from the practical experience of the authors (clinical as well as methodological experts). We tested the checklist on two occasions when performing systematic reviews [42,43].

The systemisation of the criteria has been carried out using the "PICOS" categories (Population, Intervention, Control, Outcome, Setting), and by using the assessment categories regarding internal validity, external validity, model validity, and general study quality. In the following only the essential aspects of external and model validity are pursued.

### Definitions

◆ The term "internal validity" (IV) refers to the "confidence that the trial design, conduct, and analysis has minimized or avoided biases in its treatment comparison" [44] and is considered as "a measure of the strength of the association between exposure or intervention and outcome within a study" [9]. Internal validity relates to all comparisons made between the test intervention and the controls, not only in RCTs.

◆ The term "external validity" (EV) refers to generalisability (i.e. the extent to which the effects observed in a study truly reflect what can be expected in a target population beyond the people included in the study [2]), which includes the possibility to transfer and apply study results to a distinct population/decision and patient's situation. The most important criteria are conformity with everyday practice and clinical relevance. Difficulties in assessing EV derive from the point that the target population and target setting – for which the study claims to be valid – is commonly not described explicitly. The so-called everyday practice or everyday efficacy is sometimes hard to define as well. Moreover this outer context may change with time (e.g. mutation of infectious agents).

A good external validity in a sense of an adequate reflection of reality (is it correct?) does not necessarily mean a good (external) utility in a sense of a useful reflection of reality (what is it good for? e.g. in terms of patients' quantity and quality of life)

◆ The term "model validity" indicates the concordance between the study design and an ideal setting, e.g. the "state of the art" procedures (see Wein [37]).

The differentiation between EV and MV is not very wide spread. The distinction between everyday conditions and ideal conditions becomes important when switching the focus from the confirmation of an efficacy in principle to the question of a broader application of an intervention ("everyday effectiveness"). In the first, it is important to have ideal conditions such as well trained and highly experienced therapists, a population which is supposed to be very sensitive to the intervention, outcome parameters that reflect the intervention effect the best and a setting that ensures an optimal compliance (e.g. application of a medication by intravenous infusion in a hospital instead of oral application at home). In the second, factors such as practicability of an intervention (e.g. by GPs), accessibility for patients to an intervention, frequency of concomitant diseases and medications, which may be contraindications to the intervention, patients' and therapists' preferences and others become more important.

It is often assumed that statements or conclusions concerning the efficacy are solely related to IV, and EV can only be used to generate statements concerning the extent of validity (or limits of generalisation). However, we take the position that insufficient MV and also EV can distort statements concerning the efficacy/effectiveness. For this reason, the possibilities of bias, in analogy to the IV, have been carried over into the categories of EV and MV. The principle of this extrapolation is shown in table 1, where the contrasting aspects between internal and external validity in respect of the above mentioned bias factors is compiled.

**Table 1:** System of bias factors, which may affect internal and external validity

**Bias factors**		**Internal validity**	**External validity/generalisability**
**Selection bias**	Problem	Treatment and control group are different, e.g. differences in age, severity of disease	Study group and "target group" are different, study group is not representative, e.g. differences in age, severity of disease
	
	Solution	Randomisation, matched pairs	Identification (and adjustment as far as possible) of relevant epidemiological factors, e.g. by comparison with patients who have not consented in the study
	
	False negative/positive results may occur:	–: relevant distinctions/subgroups unknown → levelled outcome +: e.g. treatment group with more "responders"	+/–: e.g. study group with more advanced disease (university hospital) +: e.g. study group without concomitant diseases (better prognosis than "usual" patients)
	
	Key questions	Is randomisation adequate? Are (known) relevant factors distributed equally?	Are relevant epidemiological factors taken into account?

**Performance bias**	Problem	Apart from the intervention tested, groups are treated differently	Study treatment does not reflect the actual variability in managing disease and patients' problems
	
	Solution	Blinding, documentation of possible differences, change to open label design (COLA design)	Treatment as realistic as possible with individualised modification if necessary (pragmatic controlled trials)
	
	False negative/positive results my occur:	–: concomitant therapy in control group; non compliance in verum group; +: concomitant therapy in verum group	+: high compliance (e.g. in hospitals); highly specialised therapists; high dosages of medication –: relevant context factors are missing (patient-therapists relationship, accessibility to therapy); inexperienced therapists; low dosages of medication
	
	Key questions	Is blinding adequate and checked? Are concomitant interventions documented?	Are realistic interventions applied which are carried out by physicians in everyday practice?

**Attrition bias**	Problem	Drop out rates between groups are different or that large that analysis is not reliable any more	Drop out rates between study group an target group are different, e.g. different compliance and/or motivation
	
	Solution	Intention to treat analysis (note: drop out rates > 10 % have a high risk of bias)	Compliance control and assessment
	
	False negative/positive results may occur:	– : intention to treat analyses +: drop out rates are higher in treatment group (with per protocol analyses)	–: drop outs due to adverse effects (and intention to treat analysis) +: drop outs due to ineffectiveness of therapy (and per protocol analysis)
	
	Key questions	Is the drop out rate documented? Are adequate analyses performed?	Are the reasons for dropping out documented? Do the reasons for dropping out have an impact on the assessment of compliance, effectiveness or safety?

**Detection bias**	Problem	Differences in the perception of outcome parameters between groups and within the the course of the study	Outcome parameters and/or length of follow up have no practical relevance to patients' problems
	
	Solution	Blinding of assessors; if blinding is not possible: assessment of two independent persons; objective parameters	Selection of clinically relevant and generally available outcome parameters; adequate length of follow up
	
	False negative/positive results may occur:	–/+: inadequate blinding and respective expectations by assessors	–: outcome parameters do not reflect actual improvement; inadequate follow up +: significant but irrelevant outcomes
	
	Key questions	Blinding procedures of assessors adequate? Independent assessors?	Are outcome parameters, length of follow up and detected differences relevant?

## Results

The checklists for assessing external and model validity are compiled in table 2 and 3.

**Table 2:** Questions for assessing external validity (EV)

**Categories**	**Items**	**+**	**(+)**	**-**	**c.b.e.**
Study population – assessment of selection bias (related to EV)	• To what extent do the inclusion and exclusion criteria (where relevant, other selection criteria) define the "everyday or target population" of the intervention?	☐	☐	☐	☐
	
	• Does the applied diagnostic procedure reflect everyday conditions and the everyday possibilities (access, necessity) respectively?	☐	☐	☐	☐
	
	• Are the diagnostic procedures and evaluations performed by persons with similar qualification and experience as in everyday practice?	☐	☐	☐	☐
	
	• Does the study population reflect the everyday population in terms of:	☐	☐	☐	☐
	○ Severity of the illness	☐	☐	☐	☐
	
	○ Duration of illness	☐	☐	☐	☐
	
	○ Intra-individual variability	☐	☐	☐	☐
	
	○ Age	☐	☐	☐	☐
	
	○ Gender	☐	☐	☐	☐
	
	○ Further socio-demographic characteristics	☐	☐	☐	☐
	
	○ Therapy preferences and expectations	☐	☐	☐	☐
	
	○ Symptoms of side effects of the interventions	☐	☐	☐	☐
	
	○ Accompanying illnesses	☐	☐	☐	☐
	
	○ Accompanying medication	☐	☐	☐	☐
	
	○ Further prognostic or therapy relevant parameters?	☐	☐	☐	☐
	
	• Has the structural similarity between the study and the everyday population or target population been tested?	☐	☐	☐	☐

Intervention und control – assessment of performance bias (related to EV)	• Does the preparation (medication, other medicinal products, other kind of interventions) reflect the usual treatment?	☐	☐	☐	☐
	
	• In case of medication, does the dosage reflect the usual treatment? (Is dose modification possible?)	☐	☐	☐	☐
	
	• Does the type of administration reflect the usual treatment?	☐	☐	☐	☐
	
	• Does the intervention duration reflect the usual treatment duration?	☐	☐	☐	☐
	
	• Are the permitted accompanying treatments the usual accompanying treatments?	☐	☐	☐	☐
	
	• Does the study situation reflect the common treatment situation?	☐	☐	☐	☐
	
	• Are the interventions carried out by therapists with similar qualifications and experience as in everyday practice?	☐	☐	☐	☐

Outcome measurements, results and evaluation – assessment of detection and attrition bias (related to EV)	• Are the chosen outcomes practice and patient relevant? (E.g. no surrogate parameter, are individual therapy goals defined?)	☐	☐	☐	☐
	
	• Were the following important outcomes considered: quality of life, subjective health, patient's general evaluations, compliance, reasons for dropout, use of accompanying treatments, rebound effect following termination of treatment (or, for example, symptom deceit)?	☐	☐	☐	☐
	
	• Are the test procedures used in usual practice?	☐	☐	☐	☐
	
	• Are the tests and evaluations performed by persons with similar qualifications and experience as in every day practice?	☐	☐	☐	☐
	
	• Are the differences clinically relevant?	☐	☐	☐	☐
	
	• Were sufficient data collected to cover the intra-individual variability?	☐	☐	☐	☐
	
	• Do the test conditions reflect the everyday practice?	☐	☐	☐	☐
	
	• Does the dropout rate reflect everyday experience? Are the reasons for dropout registered (e.g. adverse effects, insufficient effect), so that the significance for the everyday effectiveness can be assessed?	☐	☐	☐	☐
	
	• Is clinical relevance considered in the conclusion?	☐	☐	☐	☐

Study design and Setting (related to EV)	• Is the research question clinically relevant?	☐	☐	☐	☐
	
	• Does the study design ensure a high EV?	☐	☐	☐	☐
	
	• Does the study setting reflect the everyday conditions?	☐	☐	☐	☐
	
	• Are the investigators the regular contact persons (e.g. GP or relevant clinic doctor, or are they at least comparable in terms of training, status, experience, preferences; does the number of contact people reflect the usual setting)?	☐	☐	☐	☐
	
	• Does the doctor/therapist-patient relationship reflect the everyday conditions (e.g. frequency of contact, constant contact person)?	☐	☐	☐	☐

+ Matches completely/is completely fulfilled

(+) Matches incompletely but sufficiently/is only partly but sufficiently fulfilled

- Does not match or matches insufficiently/is insufficiently fulfilled

c.b.e. Can not be evaluated

**Table 3:** Questions for assessing model validity (MV)

**Categories**	**Items**	**+**	**(+)**	**-**	**c.b.e.**
Study population – assessment of selection bias (related to MV)	• To what extent do the inclusion and exclusion criteria and, where relevant, other selection criteria define an optimal population with respect to the test intervention? (An optimal population will show the highest benefit from the applied intervention).	☐	☐	☐	☐
	
	• Is the applied diagnosis and/or classification relevant for the intervention?	☐	☐	☐	☐
	
	• Are relevant subgroups considered?	☐	☐	☐	☐
	
	• Does the diagnostic procedure optimally reflect the aptitude for the intervention?	☐	☐	☐	☐
	
	• Are the diagnostic procedures performed by qualified and experienced physicians?	☐	☐	☐	☐
	
	• Does the study population reflect the ideal population in terms of:	☐	☐	☐	☐
	
	○ Severity of the illness	☐	☐	☐	☐
	
	○ Duration of the illness	☐	☐	☐	☐
	
	○ Intra-individual variability	☐	☐	☐	☐
	
	○ Age	☐	☐	☐	☐
	
	○ Gender	☐	☐	☐	☐
	
	○ Further socio-demographic characteristics	☐	☐	☐	☐
	
	○ Therapy preferences and expectations	☐	☐	☐	☐
	
	○ Symptoms of the side effects of the interventions	☐	☐	☐	☐
	
	○ Accompanying illnesses	☐	☐	☐	☐
	
	○ Accompanying medication	☐	☐	☐	☐
	
	○ Further prognostic or therapy relevant parameters? (The above listed factors can influence the measurement of outcomes so that floor/ceiling-effects may occur)	☐	☐	☐	☐
	
	• (Is the structural similarity between the study population and the ideal population for the intervention tested? – A fairly hypothetical question)	☐	☐	☐	☐

Intervention und control – assessment of performance bias (related to MV)	• Is the investigational intervention the optimal treatment?	☐	☐	☐	☐
	
	• In case of medication, is the dosage the optimal treatment?	☐	☐	☐	☐
	
	• Is the application the optimal treatment?	☐	☐	☐	☐
	
	• Is the intervention duration the optimal treatment duration? (Are there signs of marketing strategies of pharmaceutical companies?)	☐	☐	☐	☐
	
	• Are the permitted accompanying treatments the optimal accompanying treatments?	☐	☐	☐	☐
	
	• Are the study conditions the optimal conditions for the intervention?	☐	☐	☐	☐
	
	• Are the interventions carried out by qualified and experienced therapists?	☐	☐	☐	☐

Outcome measurements, results and evaluation – assessment of detection and attrition bias (related to MV)	• Do the outcome parameters reflect the effects of the intervention in the best possible manner? (Also consider here floor/ceiling effects).	☐	☐	☐	☐
	
	• Do the applied test procedures best reflect the chosen outcomes of intervention effects?	☐	☐	☐	☐
	
	• Are the test conditions appropriate to optimally evaluate the intervention efficacy?	☐	☐	☐	☐
	
	• Is the length of follow-up sufficient to detect the intervention effects (including adverse effects and rebound effects following termination of the treatment)?	☐	☐	☐	☐
	
	• Is there an analysis carried out that considers the actually applied treatment interventions (PP analysis)?	☐	☐	☐	☐
	
	• Are tests and evaluations carried out by qualified and experienced examiners?	☐	☐	☐	☐
	
	• (Retrospectively, were optimal conditions given for the identification of the intervention efficacy?)	☐	☐	☐	☐

Study design and setting (related to MV)	• Does the research question reflect the optimal conditions for the intervention?	☐	☐	☐	☐
	
	• Does the study design ensure a high level of MV?	☐	☐	☐	☐
	
	• Does the study setting reflect the optimal treatment conditions?	☐	☐	☐	☐
	
	• Do the therapists/investigators have adequate experience with the intervention or the indication in question?	☐	☐	☐	☐
	
	• Do therapists/investigators and patient have a positive attitude towards the intervention?	☐	☐	☐	☐

+ Matches completely/is completely fulfilled

(+) Matches incompletely but sufficiently/is only partly but sufficiently fulfilled

- Does not match or matches insufficiently/is insufficiently fulfilled

c.b.e. Can not be evaluated

To answer the questions regarding the EV and the MV, certain information should be collected (table 4).

**Table 4:** Information to be collected for ascertaining reference values for external validity (EV) and model validity (MV)

**Categories**	**Items**
Study population	• Characterisation of the routine and optimal indication and population, respectively for the therapy (where appropriate with modifications); for what kind of patients with the same or similar conditions are alternative therapies favoured?
	• Which diagnostic tests are routinely used in the diagnostic procedure?
	• Where possible, typical and optimal patient characterisation in relation to:
	○ Severity of the illness
	○ Duration of illness
	○ Intra-individual variability
	○ Age
	○ Gender
	○ Further socio-demographic characteristics
	○ Therapy preferences and expectations
	○ Symptoms of adverse effects of the interventions
	○ Accompanying illnesses
	○ Accompanying medication
	○ Further prognostic or therapy relevant parameters?
	• Therapy expectation for the "everyday" or "optimal" population (are these discussed with the patients)? Data on course of illness without treatment?
	• Other than the aforementioned characteristics?
	• Which patients are not suitable for the intervention and why?

Intervention und Control	• Which are the most commonly prescribed or best effective drugs/interventions for the indication/illness in question (gold-standard), and how do they differ from the investigational drug/intervention? When will the study intervention be selected, when the therapy alternative?
	• In case of medication, usual dosage of the therapy; is this related to the optimal effect? (Or is this a reduced dose due to adverse effects?)
	• Routine method of application of the therapy, is this also the optimal application?
	• Routine therapy duration? (Or by continuous application: after which period of time is a treatment-free interval considered? When are possible changes to alternative medications considered?)
	• Usual accompanying treatments in the therapy of the illness?
	• Usual accompanying therapies for commonly associated illnesses? Are interactions with investigational or similar drug/intervention known?
	• Are typical characteristics of the drug/intervention known (e.g. taste, odour, or local irritation following application, whereby the intervention can become "un-blinded")?
	• Known adverse effects of the intervention?
	• Context factors of the usual or optimal interventions treatment (e.g. individual modification of therapy, therapy expectation of the physician and patient, kind of medical care, accessibility to the therapy)?

Outcome measurements, results and evaluation	• What are the relevant outcomes for the patients (or their relatives) in practice?
	• Which parameters are routinely assessed for the progress evaluation of the illness/indication?
	• Which outcome measurements best reflect the intervention's efficacy?
	• What are the routine assessment procedures for the chosen outcome measurements and their clinically relevant threshold values?
	• Is intra-individual variability in illness progress accounted for?
	• What are the routine conditions for the test procedure?
	• What are the optimal conditions for the representation of the intervention efficacy?
	• When will the intervention effect become apparent (earliest, latest)?
	• When are adverse or rebound effects expected after termination of the application?
	• Estimate of the compliance for the intervention (in comparison to alternative therapies), reasons for non-compliance or dropouts, for what reasons is the intervention terminated, after what average time period?
	• (Questions regarding the model validity of individual studies may at best be answered by topic experts)

Study design and setting	• What are the clinically relevant research questions?
	• Characteristics of best cases/settings and worst cases/settings for the treatment?
	• Description of the standard treatment setting (and variability), and optimal setting?
	• Are there specifically relevant factors for the indication or therapy?
	• Which physicians have the most experience with the intervention or the indication (practice/clinic, speciality, level of training, experience)?
	• Typical first and subsequent contact with the intervention from the patient's perspective?
	• Time and effort for medical care in routine and optimal situations? Other relevant context factors (e.g. trust in therapy and/or physician)?
	• Time and effort for routine and optimal documentation?
	• (For hypothesis testing without comparison group: Is the reference value clinically relevant?)
	• Where appropriate: Consult specialists for an evaluation of the closeness to practice of individual studies or other factors concerning EV and MV.

**Figure 1:**
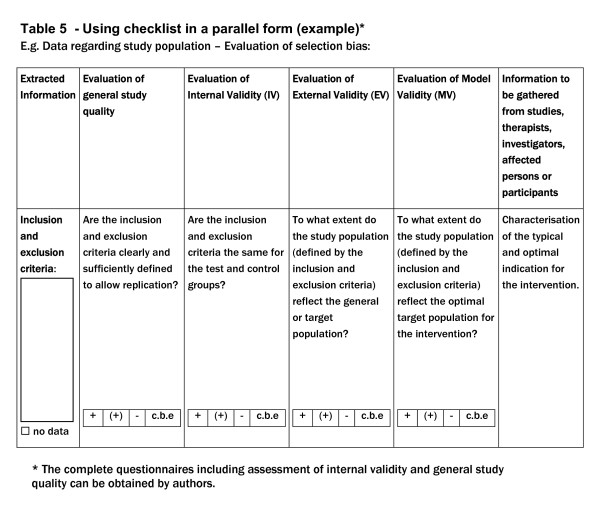
Table 5

Beside the use in a sequential form as seen in table 2 and 3 one can also consider a parallel form (Figure 1).

The complete questionnaires (including those for internal validity and general study quality) can be obtained by authors.

## Discussion

With this compilation of important parameters for MV and EV we propose a checklist, which on the one hand can be used for planning and on the other hand for evaluation of clinical studies. We would like to stress that adjustments or even more extensive modifications can be necessary according to the concrete questions of interest. According to our experience in most studies only a few aspects are crucial for the quality of the validities, while others are only of marginal importance. Some studies may lose their significance and relevance due to one single crucial error while other studies will not despite several but less important parameters judged as insufficient. Establishing and using scores harbours the risk of pseudo-accuracy. Therefore we rather suggest a descriptive evaluation, where scores should only be used to verify one's own evaluation.

The parameters necessary for the evaluation of the EV and MV should be discussed for each research question and application individually. The validity of data needed to determine these criteria is another crucial point. We recommend to avail oneself of the principles of maximal and minimal contrasting as they have worked out well in qualitative research strategies: to look for perspectives on a chosen item as different as possible for maximal contrast (e.g. therapists, methodological experts, patients and relatives in respect to a special disease) and to look for at least 2 representatives of each perspective for minimal contrasting. (General perspectives would be those of bearing responsibility for a decision/deed, implementing it and being affected by it). Gathering the data can be done by questionnaires or structured interviews using the items of the checklists (table 4). As for the validity of these collected data it appears adequate from a pragmatic point of view to consider congruent answers as reliable and deduct the reference data from them, whereas incongruent answers require further analysis. Published data on epidemiology or about clinical studies should be included in the process of compiling reference data. It can be expected that with more thorough consideration of the criteria for EV and MV in study designs future data will have higher validity. As a further result of systematic collected data according to a checklist gaps of knowledge may become evident that could possibly be addressed by additional investigations or studies.

When applying criteria of IV, EV and MV mostly not all of these criteria will be fulfilled to the same extent. That means that studies will usually not be "optimal". Which aspect will be prioritised depends on the question of the study. In the systematic reviews we performed using the checklist for EV and MV [41,42] we identified other studies as being of high quality than using criteria of IV alone. Most of the studies only considered aspects of IV. In one review [42] the assessment of effectiveness changed in favour to the treatment when prioritising aspects of external validity.

An explanatory study investigating causal connections (e.g. efficacy) will focus on IV although EV and MV should not be neglected, whereas in health-care research the presented aspects of EV should be of primary importance. To obtain a high IV or MV the study population should be as homogeneous as possible, while in evaluating EV it is of great interest to what extent the intervention is also applicable among a heterogeneous population and under heterogeneous conditions, particularly with concomitant diseases and co-medications. Homogeneity within a group is usually attained by restrictive inclusion and exclusion criteria, homogeneity and comparability between groups by randomisation. With regard to IV it is the best method since randomisation is the only adequate means to reduce the risk of the unequal distribution of unknown confounding factors. EV is, as presented above, with high probability affected by the randomisation [11,14,15].

Furthermore, it can be assumed that the MV (which may be already distorted through the selection process alone) will be impaired since the ethically and methodically requested prerequisites for the randomisation – the so-called equipoise, i.e. the unbiased position of the investigator in respect of intervention and control – may not be sufficiently fulfilled. Great experience (high MV!) presumably comes along with therapist's preference for a certain intervention, which may interfere with the required neutrality towards the treatment options. To consider the therapy preferences of the physician and the patient within a study corresponds to a high MV and EV.

A study design satisfying the need of IV and EV could be a 4-arm study, in which two arms represent the respective preference for the test or the control intervention – being an open and not blinded intervention – while the other two arms representing the randomised, blinded trial with genuine equipoise. Further possibilities are studies with change-to-open-label (COLA) design [24,45] or propensity score analyses; a very high EV is also associated with the formation and evaluation of medical registers. A particular ethical problem regarding the equipoise exists in placebo controlled studies, where patients should in principle have the confidence to receive the best therapy and not solely to be used for the gain of knowledge (see in addition also Horrobin [46]). Strictly speaking, to warrant the equipoise only physicians who consider the treatment-free "therapy" or placebo application to be a justified therapeutic option should carry out placebo-controlled studies.

An intervention within a study may be altered, e.g. by individual dose modification or accompanying treatments, satisfying the needs of the everyday life reality. The intervention itself is seen as needed, but not necessarily as sufficient in the individual case. Study designs suitable for these settings are "pragmatic controlled clinical trials" [21,31,32], which are, however, deficient in IV.

Naturally, the question arises whether the expenditure to apply the presented checklist is justified. First of all we want to emphasise that from this checklist's systematised compilation not all aspects will need to be addressed for a particular research question and that they also are, though deliberately, partly redundant. Therefore, the expenditure in the actual application will be lessened.

When using the checklist in the process of study planning to decide which aspects should or should not be considered the already strenuous effort of this process may only slightly increase. When applying the checklist for the evaluation of clinical studies, however, the expenditure is much more time consuming compared to other, at the present used, evaluation methods (e.g. Jadad score). However, it appears to be justified to do so considering the expenditure in regard to personnel and funding and in regard to the (psychological) strain for patients to participate in a study. Studies may otherwise be excluded from a further evaluation in a meta-analysis or a systematic review and may not be considered for generating guidelines for more or less formal reasons; or they will be included due to their high IV despite a low EV. Particularly with respect to the generation of guidelines, which have or should have a large influence on the decisions about the therapy, the relevant factors can not be weighted carefully enough. Furthermore, it could be expected that the acceptance of guidelines will be substantially higher in clinical application, if in the planning of the studies aspects of external validity were already considered.

## Conclusion

IV, EV and MV are important parameters when assessing clinical studies. Since EV and MV tend to be often neglected we have created a comprehensive checklist addressing the different types of validity. The checklist can be applied to both, planning and evaluating clinical studies and can be modified according to the actual research question. It is our hope that this checklist will enhance the consideration of particularly EV and MV in clinical trials.

## Competing interests

The author(s) declare that they have no competing interests.

## Authors' contributions

Details of contributors:

GB: conception, design, analysis, interpretation, writing; SMB and UW: conception, revising article; GSK: analysis, revising article; AM: interpretation from clinical point of view, revising article; HCV: interpretation from general practitioner's point of view, methodological aspects, revising article; SG: interpretation from qualitative researcher's point of view, writing article; PFM: conception, revising article. All authors read and approved the final manuscript.

## Pre-publication history

The pre-publication history for this paper can be accessed here:


